# Integrating the United Nations sustainable development goals into higher education globally: a scoping review

**DOI:** 10.1080/16549716.2023.2190649

**Published:** 2023-03-31

**Authors:** Ángela Amorós Molina, Daniel Helldén, Tobias Alfvén, Maria Niemi, Karin Leander, Helena Nordenstedt, Carita Rehn, Rawlance Ndejjo, Rhoda Wanyenze, Olivia Biermann

**Affiliations:** a Department of Global Public Health, Karolinska Institutet, Stockholm, Sweden; b Centre of Excellence for Sustainable Health, Karolinska Institutet in Stockholm, Solna, Sweden; c The Centre of Excellence for Sustainable Health, Makerere University in Kampala, Kampala, Uganda; d Institute of Environmental Medicine, Stockholm, Sweden; e Department of Medicine and Infectious Diseases, Danderyd University Hospital, Stockholm, Sweden; f School of Public Health, Makerere University, Kampala, Uganda

**Keywords:** United Nations, Agenda 2030, sustainable development goals, higher education, sustainability education

## Abstract

**Background:**

In 2015, the United Nations adopted the 2030 Agenda for Sustainable Development, including the 17 Sustainable Development Goals (SDGs). Higher education institutions have a role in raising awareness and building skills among future professionals for implementing the SDGs. This review describes how the SDGs have been integrated into higher education globally.

**Objectives:**

Determine how have the SDGs been integrated into higher education globally. Describe the differences in the integration of the SDGs in higher education across high-income countries (HICs) and low- and middle-income countries (LMICs).

**Methods:**

Following a scoping review methodology, we searched Medline, Web of Science, Global Health, and Educational Resources Information Center, as well as websites of key institutions including universities, identifying peer-reviewed articles and grey literature published between September 2015 and December 2021.

**Results:**

We identified 20 articles and 38 grey literature sources. Since 2018, the number of publications about the topic has been increasing. The SDGs were most frequently included in bachelor-level education and disciplines such as engineering and technology; humanities and social sciences; business, administration, and economics. Methods of integrating the SDGs into higher education included workshops, courses, lectures, and other means. Workshops and courses were the most frequent. The methods of integration varied in high-income countries compared to low- and middle-income countries. High-income countries seemed to follow a more academic approach to the SDGs while low- and middle-income countries integrate the SDGs with the aim to solve real-world problems.

**Conclusion:**

This study provides examples of progress in integrating the SDGs into higher education. Such progress has been skewed to high-income countries, bachelor-level initiatives, and certain disciplines. To advance the integration of the SDGs, lessons learned from universities globally should be shared broadly, equitable partnerships formed, and students engaged, while simultaneously increasing funding for these processes.

## Introduction

In 2015, all the member states of the United Nations (UN) adopted the 2030 Agenda for Sustainable Development, including the 17 Sustainable Development Goals (SDGs) (Appendix I) [1]. The SDGs are aimed at ending poverty; developing strategies for improving health, social, and economic inequalities; promoting economic growth; improving education; and enhancing environmental health for all countries [2]. Higher education institutions have a role in implementing the SDGs, even though ‘higher education’ and ‘tertiary education’ only appear twice in the UN’s Global Indicator Framework for the Sustainable Development Goals and targets of the 2030 Agenda for Sustainable Development [3,4]. For example, higher education institutions produce knowledge and raise awareness about the SDGs among their students [5,6]. Higher education institutions also provide students with the necessary skills needed to implement the SDGs, such as strategic vision, design-thinking, social responsibility, problem-solving, anticipatory skills, and inter-disciplinary collaboration [5,7]. Therefore, the integration of the SDGs in higher education can provide future professionals with the necessary knowledge, tools, and skills to successfully address the complex interrelated challenges of the future including through reciprocal learning between high- and low and middle-income countries as well as interdisciplinary and multidisciplinary learning and problem-solving. For instance, to ensure the environmental sustainability of future constructions, a future civil engineer should learn about the SDGs [5,6,8].

In the context of this study, we have defined ‘integration’ as mandatory or elective courses, workshops, lectures and other activities which can vary between HICs and LMICs [7]. However, there is currently a knowledge gap on how the SDGs have been integrated into higher education [9]. Filling this gap is key to taking stock and accelerating the integration of the SDGs in higher education thus contributing to SDGs progress [10,11]. As a response to this lack of a synthesised understanding of the integration of the SDGs in higher education, we conducted a scoping review with two main research questions: *How have the SDGs been integrated into higher education globally? What are the differences in the integration of the SDGs in higher education across high-income countries (HICs) and low- and middle-income countries (LMICs)?*

## Methodology

Due to the complex nature of the topic and the wide range of studies and sources that might be relevant to the research questions, a scoping review method was selected. The scoping review followed the methodological framework by Arksey and O’Malley [12]. We used the Preferred Reporting Items for Systematic reviews and Meta-Analyses extension for Scoping Reviews (PRISMA-ScR) checklist to guide the reporting [13–15]. The completed checklist is available in Appendix II.

### Data sources and search

We searched MEDLINE, Web of Science, Global Health, and Educational Resources Information Center (ERIC) for peer-reviewed articles from January 2015 (the year when the UN adopted the 2030 Agenda) to December 2021.

The search strategy for the included databases, which we developed in collaboration with the Karolinska Institutet Library, is included in Appendix III. The latter was standardised but adapted to fit the other database and grey literature searches. We identified relevant grey literature by reviewing reports by the UN [16], the United Nations Educational, Scientific and Cultural Organization (UNESCO) [17], and the International Association of Universities [18]. Moreover, we searched for higher education institutions’ websites.

To select higher education institutions, we first identified five top-level institutions per geographical region (Africa, Asia, Europe, North America, South America, and Oceania) from three university rankings [19–21]. We imported the results of the searches into the study selection software Rayyan [22].

### Selection, screening, and charting

We selected articles and grey literature according to the following inclusion criteria: focus on the SDGs as the main topic, publication between 2015 and 2021, higher education as the setting, and explicit mention of how the SDGs have been integrated into higher education. We used the following exclusion criteria: focus on the concept of sustainable development more broadly without focus on the SDGs, no specification of the country of implementation, and language other than English or Spanish. We determined ‘integration’ based on the description of courses, workshops, lectures and other activities, while we did not apply any score or scale to measure the integration of the SDGs.

One author (AAM) initially removed duplicates using the Rayyan software and then screened the articles first by title, second by abstract, and finally by full text. The grey literature sources were also screened according to the inclusion and exclusion criteria. AAM then charted the data based on different variables: country, study programme (e.g. healthcare, education, and engineering), method of integration of SDGs (e.g. workshops, courses, and lectures), partnership development (e.g. governments, other universities, and communities), higher education involvement in policy development, financing of SDGs integration, and future actions. The countries were classified as HICs and LMICs according to the World Bank classification (2021) [23]. OB and DH reviewed the data charting to ensure the relevance and validity of the data extracted. OB, DH, and TA were consulted during all stages of the scoping review.

### Patient and public involvement

We have not involved patients or the public in the development of the research question, study design, and conduct.

## Results

**Figure 1.**
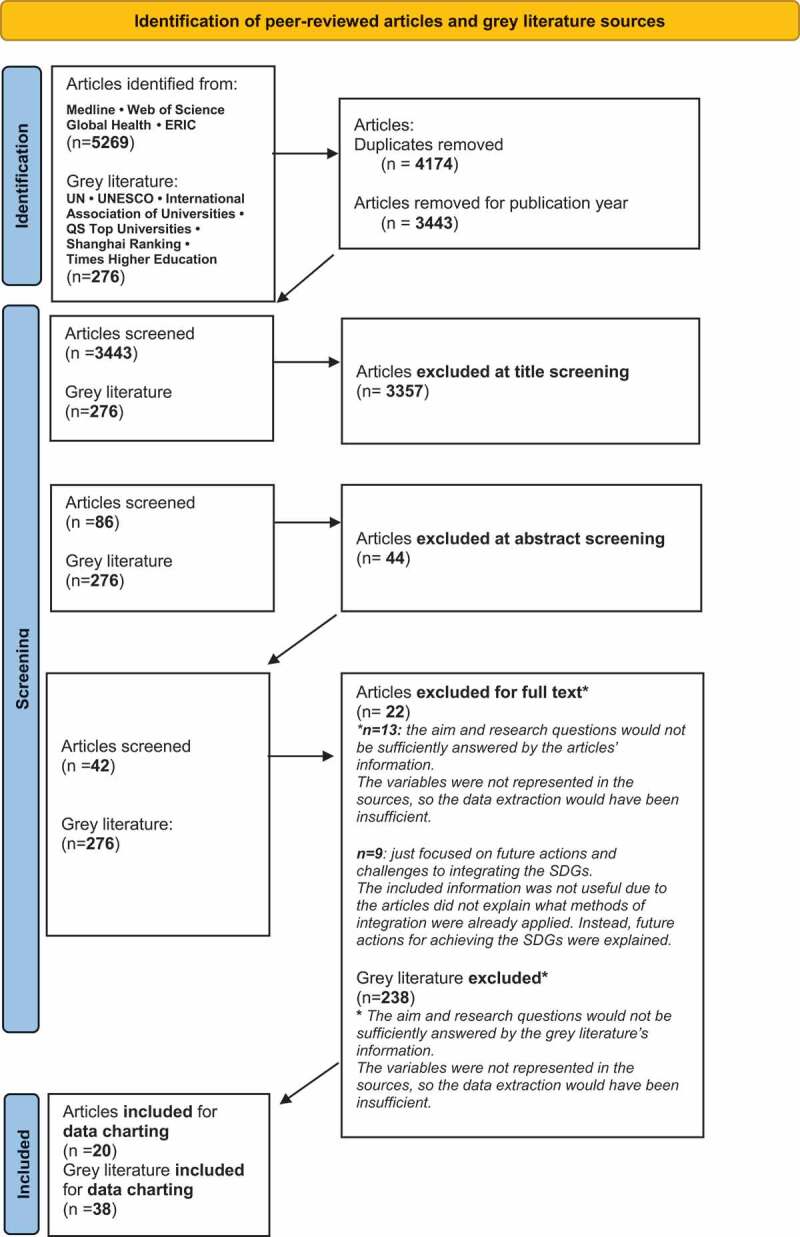
Prisma flowchart for study selection.

We identified 20 out of initially 5269 peer-reviewed articles [3,24–42] and 38 out of 276 grey literature sources through this scoping review [43–76,76–79] (Figure 1). The reasons for excluding articles and grey literature sources are provided in Figure 1. The included publications are heterogeneous in detailing the results and their in-depth findings.

### Characteristics of the included publications

Of the 20 publications, the greatest number of them were published in 2021 (40%) [24,26,27,29,30,35,42,80]. In general, the number of articles published has increased since 2018. The studies were equally distributed between mixed methods studies (35%) [3,28,30–32,36,39], quantitative (30%) [24,33–35,41,42], and qualitative studies (35%) [25–27,29,37,38,40]. Moreover, 40% of the articles were published in the International Journal of Sustainability in Higher Education [3,25,29,32,33,40–42]. Regarding the country of origin, 36% of the articles were published in LMICs. Most grey literature was published in 2022 (47%) [49,50,53,56,58,60–65,67–71,74,78], mostly on university websites (65%). Furthermore, 28% of the grey literature sources were from LMICs.

### Educational levels and disciplines

We found examples of the integration of the SDGs at all education levels (bachelor, postgraduate, master, and PhD); however, according to the peer-review articles and the grey literature, the SDGs were most frequently integrated at the bachelor level (28%) [28–32,35,36,38,42,47,55,63,68,70,71,73]. Table 1 shows as an example of the findings from the publications how the SDGs have been integrated across educational levels according to the peer-reviewed articles. The methods of integration that have been identified in the literature can be categorised into workshops, courses, lectures, and other activities (such as degree topics, seminars, and webinars).

**Table 1.** Overview of SDG integration across educational levels.

Number	Reference	Country	Educational level	Educational discipline	Method of integration	Content
1	Albareda, et al. [32]	Spain	Bachelor	Law and Political Science, Business and Administration, Dentistry, Humanities, Architecture, Audiovisual Communication and Public Relations and Journalism, Primary Education and Preschool Education, Medicine, Nursing, and Physiotherapy.	Others	Methodological strategies to apply to real-world problems and to work together with stakeholders.
2	Aleixo, et al. [33]	Portugal	Bachelor and master	Engineering, Life and Health Sciences, Natural and Environmental Sciences, Social Sciences, and Humanities.	Course and lectures	Focus on linking theory and practice in a community-oriented approach, using different methodologies such as case studies, lecturing, role-playing, etc.
3	Anasi, et al. [34]	Nigeria	No information	No information	Others	Academic libraries and librarians capture, analyse, organise, store, and share internal and external information resources for effective knowledge exchange among users through the Online Public Access Catalogue which is indispensable in the knowledge transfer for the SDGs achievement.
4	Blasco,et al. [24]	Spain	No information	No information	Others	Investment in research for developing the integration of SDGs in society.
5	Chaleta, et al. [35]	Portugal	Bachelor	Social Sciences	Course	Focus on SDG 4 with an accentuation on inequalities, the rise in unemployment, and the possibility of another economic crisis.
6	Chang Ya-Ching, et al. [36]	Taiwan	Bachelor	Humanities and social sciences, Law, Management, Sciences, and Engineering.	Course	Focus on teachers from higher educational institutions to prepare the courses syllabus.
7	Cottafava, et al. [37]	Italy	No information	No information	Workshop	Application of managerial skills to create innovations through the development of business projects. Made of modular blocks (fundamental understanding of SDGs and development of managerial reasoning through the practical use of SDGs for solving real-world problems) that allow replicability and scalability.
8	Da Wan, et al. [80]	Malaysia	Bachelor	No information	Others	Top-down integration from the government from the creation of working committees: inclusivity, well-being, human capital, environment and natural resources, and economic growth.
9	Expósito, et al. [38]	Spain	Bachelor	Tourism, engineering, architecture, social sciences, psychology, chemistry, law, computer science, philosophy, economy, and nursing.	Course	Enhance the professional development of university teachers in the SDGs field. Understanding the complexity behind sustainability and its representation in the SDGs. Identifying the main characteristics of the local, regional, and global contexts that affect higher education. To apply the contents of the course in reorienting the design of a university subject course towards sustainability.
10	Ezquerra-Lázaro, et al. [27]	Spain	Bachelor, master, and PhD	No information	Workshop and others	Workshops related to how higher education institutions and research community transform the university in a sustainable way. Other methods of integration are based on SDGs seminars to prepare the faculty and research community for change towards the UN’s 2030 Agenda.
11	Gómez, et al. [42]	Spain	Bachelor	Engineering	Workshop, course, and others	Workshops about the analysis of the SDGs’ presence in public and private entities in the civil engineering sector. Orientation of the core courses (math, structural engineering, economics, etc.), with the SDGs scope. Other methods are to include the SDGs perspective in an annex in the bachelor thesis project and incorporate a critical reflection into their bachelor’s thesis, describing the contribution or relationship of their thesis with the 2030 Agenda and the SDGs.
12	Hansen, et al. [30]	Florida	Bachelor	No information	Course and others	The course is about the SDGs and their integration into campus and the community. Other types of methods of integration are based on webinars and communication boards about sustainability, as well as free courses and a global competency test that covers SDGs and sustainability literacy.
13	Leal, et al. [39]	Europe	Bachelor, master, and PhD	No information	Lectures and others	Based on research activities, living labs, and simulations of behavioural changes.
14	Lovren, et al. [40]	Serbia	Bachelor, master, and PhD	Architecture, Philosophy, Security studies	Course	three perspectives of sustainability: social well-being, economic prosperity, and environmental health. Importance of addressing the interrelation between humans and the environment in a holistic perspective. Studying environmental challenges and educational solutions rely on sustainability.
15	Mawonde, et al. [3]	South Africa	Bachelor, master, and PhD	Agriculture and Environmental Sciences, Economic and Management Sciences, Engineering and Technology	Workshop	Development of community-based interventions according to necessities and within the SDGs scope.
16	Petillion, et al. [31]	UK	Bachelor	Chemistry	Course	Case studies, guided-inquiry assignments, online quizzes, and exams are developed. The content is determined using the SDGs as a thematic framework for how chemicals are beneficial or harmful to society. Method also offered in master’s and PhD.
17	Purcell, et al. [25]	UK	No information	No information	Courses and others	Courses are related to investigating solutions to real-world sustainability challenges. Other methods of integrating the SDGs are about developing real-world learning and research opportunities for students and higher education institutions.
18	Ramirez, et al. [41]	Mexico	Bachelor, master, and PhD	Engineering, Computer Science, Nanotechnology, Biotechnology	Others	Development of transversals and disciplinary competencies, through real problematic bonded challenge resolution and by proving their knowledge and dominance through learning evidence. Development of research projects related to environmental sustainability (sanitation and hygienic environment).
19	Togo, et al. [29]	Zimbabwe	Bachelor	Soil Science, Agricultural Engineering, Mechanical Engineering, Civil Engineering, and Environmental Sciences.	Others	Projects development linked with the community to evaluate the impact of the SDGs in local and global contexts.
20	Useh [26].	South Africa	Postgraduate, master, and PhD	Science, Technology, Engineering, Mathematics	Others	Transdisciplinary research which focuses on social needs.

Table 2 provides an overview of the peer-reviewed articles and grey literature of the disciplines that have integrated the SDGs and their distribution across LMICs and HICs. Disciplines that have integrated the SDGs more frequently include engineering/technology and humanities/social sciences, compared to, for example, healthcare. The integration of the SDGs in all disciplines was more common in institutions in HICs compared to LMICs. In some cases, multiple disciplines at one university integrated the SDGs, e.g. in Zimbabwe, the SDGs were integrated into Soil Science, Agricultural Engineering, Mechanical Engineering, Civil Engineering, and Environmental Sciences [30].

**Table 2.** Disciplines that integrated the SDGs and the distribution globally.

Disciplines	N	HICs	LMICs
Engineering/Technology	13	57%	43%
	[3,26,33,36,38,41,42,47,49,52,55,64,68]		
Humanities/Social Sciences	9	80%	20%
	[32,33,35,36,38,53,68,70]		
Business/Administration/Economics	10	80%	20%
	[3,32,38,61,63,69,70,73,74]		
Environment/Agriculture	5	67%	33%
	[3,33,53,54,61]		
Architecture	5	80%	20%
	[32,38,40,55,79]		
Law	4	100%	0%
	[32,36,38,58]		
Chemistry	2	100%	0%
	[31,38]		
Healthcare (incl. medicine, nursing, physiotherapy, and dentistry)	5	100%	0%
	[32,33,53,61,70]		
Philosophy	2	50%	50%
	[38,40]		
Journalism/Communication	1	100%	0%
	[32]		
Education	1	100%	0%
	[32]		
Tourism	1	100%	0%
	[38]		
Arts	1	100%	0%
	[53]		

### Methods of integration of the SDGs globally

We identified a variety of methods of integrating the SDGs into higher education globally, i.e. through workshops, courses, lectures, and others, which we outline in the following.

Workshops in HICs often linked the SDGs to the job market [29,33,80], e.g. teaching students in Italy managerial skills to develop business innovations within the SDG framework [37]. Other workshops focused on the increase of environmental sustainability at the university [27,42]. In examples from LMICs, workshops in South Africa revolved around the sustainable development of community-based interventions according to societal needs, sustainably solving environmental challenges for society and facilitating economic growth [3]. In Mexico, workshops focused on sustainable development through the support and empowerment of women [64].

In terms of courses, students in HICs developed projects for the integration of the SDGs on campus and in the community. In Portugal, students were taught about raising awareness of unemployment and other social inequalities [35]. In Florida (United States of America), research projects were oriented to integrate sustainability into university campuses and the community [30]. In contrast, courses in LMICs were focused on entrepreneurship in sustainability, environmental health, and raising awareness in the surrounding community about the SDGs. In Jamaica, students learned to support their community by critically analysing the relationship between individuals, different communities, and the environment; being able to apply a sustainable development focus by the evaluation of the main three pillars of sustainable development: economic, social, and ecological. In this case, the students would develop a commitment with their actions towards the community and be able to create future sustainable and healthy societies [77].

We found limited information regarding the use of lectures on the SDGs in LMICs. At a university in China, students learned about strategic resources for businesses to maintain sustainable innovation capability and to win a competitive advantage [57]. In HICs, e.g. in Denmark, higher education institutions included lectures on the need to improve gender equity and promote the sustainability of food systems [61]. These lectures about topics related to sustainability can be directly related to diverse SDGs, such as SDG 2 ‘Zero hunger’ and SDG 5 ‘Gender equality’. In Switzerland, we found a lecture series about how universities can contribute towards achieving SDGs in collaboration with other stakeholders [59]. Other types of methods of integrating the SDGs in higher education in HICs included seminars and webinars. Higher education institutions in LMICs reported that higher education institutions utilised core stakeholders, such as libraries or working committees, to integrate the SDGs in higher education institutions [34]. Articles from Spain [24] and South Africa [3] offered examples of funded research for SDG integration, while not specifying the funding source.

## Discussion

This scoping review explored examples of how the SDGs have been integrated into higher education globally. Based on 20 peer-reviewed articles and 38 grey literature sources, the SDGs were most frequently included in bachelor-level education, compared to other levels such as doctoral level, and disciplines such as engineering and technology; humanities and social sciences; business, administration, and economics, compared to disciplines such as healthcare or education. Methods of integrating the SDGs into higher education included workshops, courses, and lectures among others. The characteristics of the methods of integration varied in HICs compared to LMICs countries. In general, we found more information from HICs compared to LMICs regarding possible modes of integrating the SDGs. In addition, we found limited information on funding sources for the integration of the SDGs in higher education, as well as for the community-based angle in higher education.

The progress on integrating the SDGs into higher education has been skewed to bachelor-level initiatives and certain disciplines, while progress across university levels and disciplines could potentially accelerate the implementation of the SDGs [81,82]. As Gómez and colleagues wrote: ‘The SDGs provide a unique opportunity for universities, allowing them to demonstrate their willingness and ability to play an active and meaningful role in the development of society and their contribution to global sustainable development’ [42]. The integration of the SDGs at all university levels could increase the proactivity of companies to increase awareness about the SDGs and adapt their organisation to a more sustainable mindset [83] incorporating strategic changes in the companies’ management by pushing their will to change [84].

Future healthcare professionals need knowledge about the SDGs to reorient health towards health promotion and comprehensive primary health care with a focus on health determinants – considering also environmental hazards due to climate change and its health impacts. Factors outside of the health sector such as climate change, inequalities, and other social determinants of health are largely captured in the SDGs. However, we found that only a few healthcare educational programmes had explicitly integrated the SDGs. As such, the integration of SDGs in all educational disciplines would be necessary [85]. Chotchoungchatchai and colleagues highlight that to achieve the health-related SDGs, primary healthcare workers must learn about multisectoral action, such as collaborating with other stakeholders locally and nationally [85,86]. This missed opportunity should thus be bridged for healthcare workers and other disciplines currently being left behind.

Methods of integration of the SDGs into higher education included workshops, courses, and lectures, among other means, which could be most impactful on students’ learning if used complementarily. According to Safari and colleagues, standard academic courses are the most used teaching method in science disciplines, while their combination with workshops and other activities is seen as more enjoyable [87]. Degree projects and lectures have been described as stimuli for creating new initiatives [37] and could be useful in complementing workshops and courses. Higher education institutions in HICs seemed to follow a more academic approach to the SDGs, for example, in Spain the ‘SDGs perspective’ is included as a critical reflection in bachelor’s theses, describing their contribution to the UN’s 2030 Agenda [42]. Meanwhile, LMICs seemed to approach the SDGs with the aim to solve real-world problems, often at the community level. For example, in Zimbabwe, higher education institutions include the SDGs through project development linked with the community to evaluate the impact of the SDGs on local and global contexts [29]. Similarly, but not as frequently found in HICs, Grindsted [88] details how students can contextualise the inclusion of the SDGs in the local context, e.g. through fieldwork and interaction with local stakeholders. Students’ engagement with local stakeholders seems crucial to contextualise the SDGs and thus make them tangible. Students’ perspectives are also key in monitoring and evaluation processes of integrating the SDGs in higher education [89].

We found more information from HICs compared to LMICs regarding possible methods of integrating the SDGs into higher education. This might be due to a lack of funding, documentation, awareness, or recognition related to the importance of integrating the SDGs in higher education. Higher education institutions could act as a catalyst for change, supporting and encouraging the integration of the SDGs in all settings. Furthermore, to increase the pace of integration of the SDGs in higher education, especially in LMICs, lessons learned on the ‘how’ of integration should be shared broadly and equitable partnerships built, creating common spaces of communication and new educational processes [27]. The traditionally asymmetrical collaboration structures and power dynamics where HIC institutions and funders direct the focus and activities of institutions in LMICs must be altered, and a reformed equitable collaboration structure between higher institutions in LMICs and HICs becomes the norm [90]. For example, the Center of Excellence for Sustainable Development (CESH) is a collaboration based on decades of mutual dynamic partnerships [91] between Karolinska Institutet (Sweden) and the Makerere University (Uganda) to support the integration of the SDGs into higher education [92]. CESH also provides tools and resources for students, researchers, professionals, and policymakers.

In the future, it would be interesting to explore shifts towards teaching and learning that include multidisciplinary, hands-on, real-life challenges and community engagement approaches. Indeed, multidisciplinary teaching and learning can be useful for developing critical analysis and problem-solving capabilities that are required for complex systems approaches to the SDGs. Teaching methods that provide students with skills that can be transferable to diverse work environments would be of added value, when teaching about the SDGs. Additionally, it is not just about what is taught but the example universities provide to future leaders. As such, it would be useful to identify examples of universities adjusting their environments to align with SDGs, e.g. sustainable environments, green environments, etc. Finally, it would be relevant to explore if there are any national-level policies, guidance, and requirements for educational institutions to integrate the SDGs. This would perhaps drive the change if such policies and requirements existed.

### Limitations

This scoping review has some limitations. Only one author (AAM) performed the screening, study selection, data charting, and analysis, which may have biased the results. Also, availability bias, publication bias and reporting bias [93,94] may have skewed the results. However, OB, DH, and TA provided feedback and guidance throughout the review process to minimise these potential biases. As the included publications did not primarily aim at describing the ‘how-to’ of integrating the SDGs in higher education, the information we extracted is incomplete, e.g. we do not know the length of the described courses, or the university points awarded, and the funding mechanisms. Moreover, due to the limited time since the SDGs were introduced, the relative lack of findings in this study might to some extent be because higher educational institutions have not had the time to integrate the SDGs in their educational plans. Furthermore, we may have missed examples of the integration of the SDGs in higher education, as their integration may not have been explicitly mentioned in relation to SDG integration, e.g. in the context of gender. An additional assessment of curricula, especially in medicine and health where we found limited information, may have provided additional information on the integration of the SDGs and related concepts, which would be interesting for future studies to assess. No critical appraisal of the quality of the included articles was done, as it is not strictly required for scoping reviews [95].

## Conclusions

This scoping review provides examples of progress in integrating the SDGs into higher education, primarily through courses, workshops, and lectures on the topic. Such progress has been skewed to high-income countries, bachelor-level initiatives, and certain disciplines. To advance the integration of the SDGs in higher education across educational levels, disciplines and countries, lessons learned from universities globally should be shared broadly, especially with regard to the ‘how-to’ of integration. Equitable partnerships should be formed and students engaged, while simultaneously increasing funding for these processes.

## Paper context

Higher education institutions have a role in implementing the Sustainable Development Goals (SDGs) by raising knowledge and awareness among their students. The SDGs can be integrated into higher education in various ways, e.g. through courses, workshops, lectures, and others. Progress on integrating the SDGs across educational levels, disciplines, and countries is needed. To accelerate the integration of the SDGs in higher education, lessons learned on the ‘how’ of integration should be shared.

## Appendix I

17 United Nations sustainable development goals

**Table:**

1. No poverty	10. Reduced inequalities
2. Zero hunger	11. Sustainable cities and communities
3. Good health and well-being	12. Responsible consumption and production
4. Quality education	13. Climate action
5. Gender equality	14. Life below water
6. Clean water and sanitation	15. Life on land
7. Affordable and clean energy	16. Peace, justice and strong institutions
8. Decent work and economic growth	17. Partnerships for the goals
9. Industry, innovation and infrastructure	

## Appendix

PRISMA-ScR checklist

**Table:**

Section	IteM	Prisma-ScR checklist item	Reported on page #
**Title**			
Title	1	Identify the report as a scoping review.	Yes
			p.1
**Abstract**			
Structured summary	2	Provide a structured summary that includes (as applicable): background, objectives, eligibility criteria, sources of evidence, charting methods, results, and conclusions that relate to the review questions and objectives.	Yes
			p.2
**Introduction**			
Rationale	3	Describe the rationale for the review in the context of what is already known. Explain why the review questions/objectives lend themselves to a scoping review approach.	Yes
			p. 3
Objectives	4	Provide an explicit statement of the questions and objectives being addressed with reference to their key elements (e.g. population or participants, concepts, and context) or other relevant key elements used to conceptualise the review questions and/or objectives.	Yes
			p. 3
**Methods**			
Protocol and registration	5	Indicate whether a review protocol exists; state if and where it can be accessed (e.g. a Web address); and if available, provide registration information, including the registration number.	Yes
			p.4
Eligibility criteria	6	Specify characteristics of the sources of evidence used as eligibility criteria (e.g. years considered, language, and publication status), and provide a rationale.	Yes
			p. 4
Information sources*	7	Describe all information sources in the search (e.g. databases with dates of coverage and contact with authors to identify additional sources), as well as the date the most recent search was executed.	Yes
			p. 4
Search	8	Present the full electronic search strategy for at least 1 database, including any limits used, such that it could be repeated.	Yes
			p. 35
Selection of sources of evidence†	9	State the process for selecting sources of evidence (i.e. screening and eligibility) included in the scoping review.	Yes
			p. 4–5
Data charting process‡	10	Describe the methods of charting data from the included sources of evidence (e.g. calibrated forms or forms that have been tested by the team before their use, and whether data charting was done independently or in duplicate) and any processes for obtaining and confirming data from investigators.	Yes
			p. 4
Data items	11	List and define all variables for which data were sought and any assumptions and simplifications made.	Yes
			p. 5–6
Critical appraisal of individual sources of evidence§	12	If done, provide a rationale for conducting a critical appraisal of included sources of evidence; describe the methods used and how this information was used in any data synthesis (if appropriate).	No
Synthesis of results	13	Describe the methods of handling and summarising the data that were charted.	Yes
			p. 4–5
**Results**			
Selection of sources of evidence	14	Give numbers of sources of evidence screened, assessed for eligibility, and included in the review, with reasons for exclusions at each stage, ideally using a flow diagram.	Yes
			p. 6–7
Characteristics of sources of evidence	15	For each source of evidence, present characteristics for which data were charted and provide the citations.	No
Critical appraisal within sources of evidence	16	If done, present data on critical appraisal of included sources of evidence (see item 12).	No
Results of individual sources of evidence	17	For each included source of evidence, present the relevant data that were charted that relate to the review questions and objectives.	No
Synthesis of results	18	Summarise and/or present the charting results as they relate to the review questions and objectives.	Yes
			p.8–13
**Discussion**			
Summary of evidence	19	Summarise the main results (including an overview of concepts, themes, and types of evidence available), link to the review questions and objectives, and consider the relevance to key groups.	Yes
			p. 14–16
Limitations	20	Discuss the limitations of the scoping review process.	Yes
			p. 16–17
Conclusions	21	Provide a general interpretation of the results with respect to the review questions and objectives, as well as potential implications and/or next steps.	Yes
			p.18
**Funding**			
Funding	22	Describe sources of funding for the included sources of evidence, as well as sources of funding for the scoping review. Describe the role of the funders of the scoping review.	No

## Appendix

Search strategy for MEDLINE database.

**Table:**

Interface: Ovid MEDLINE(R) and Epub Ahead of Print, In-Process & Other Non-Indexed Citations and Daily Date of Search: 27 Sept 2021 Number of hits: 372 Comment: In Ovid, two or more words are automatically searched as phrases; i.e. no quotation marks are needed	Field labels exp/= exploded MeSH term /= non exploded MeSH term .ti,ab,kf. = title, abstract and author keywords adjx = within x words, regardless of order * = truncation of word for alternate endings	
Database(s): Ovid MEDLINE(R) and Epub Ahead of Print, In-Process, In-Data-Review & Other Non-Indexed Citations and Daily 1946 to September 27, 2021 Search Strategy:		
**#**	**Searches**	**Results**
1	exp Sustainable Development/	1467
2	exp Education, Graduate/	91413
3	exp Universities/	45504
4	(college* or graduate educat* or higher educat* or post-secondary educat* or tertiary educat* or third-level educat* or universit*).ti,ab,kf.	530524
5	2 or 3 or 4	621056
6	(agenda 2030 or SDG or smart growth or sustainability educat* or sustainable develop* or sustainable develop*).ti,ab,kf.	10748
7	((UN or united nation*) adj3 development goal*).ti,ab,kf.	939
8	6 or 7	10977
9	1 or 8	11593
10	5 and 9	372
**Search strategy for Web of Science database**		
Interface: Clarivate Analytics Date of Search: 28 Sept 2021 Number of hits: 3750	Field labels TS/Topic = title, abstract, author keywords and Keywords Plus NEAR/x = within x words, regardless of order * = truncation of word for alternate endings	
	Note: sometimes ‘quotation marks’ are needed for single search terms to avoid automatic term mapping (lemmatisation).	
5		
**(#3) AND #4**		
Edit		
Add to Search		
3,750		
4		
**(#1) OR #2**		
Edit		
Add to Search		
69,679		
3		
**TS=((‘higher educat*’ OR ‘tertiary educat*’ OR ‘post-secondary educat*’ OR ‘third-level educat*’ OR universit* OR college*))**		
Edit		
Add to Search		
951,655		
2		
**TS=((‘UN’ or ‘united nation*’) NEAR/2 ‘development goal*’)**		
Edit		
Add to Search		
2,740		
1		
**‘agenda 2030’** (Topic) or **‘SDG’** (Topic) or **‘smart growth’** (Topic) or **‘sustainability educat*’** (Topic) or **‘sustainable develop*’** (Topic) or **‘sustainble develop*’** (Topic)		
Edit		
Add to Search		
69,351		
**Search strategy for Global Health database**		
Interface: Wiley Date of Search: 30^th^ Sept 2021 Number of hits: 203		
Database(s): **Global Health** 1973 to 2021 Week 38 Search Strategy:		
**#**	**Searches**	**Results**
1	(college* or graduate educat* or higher educat* or post-secondary educat* or tertiary educat* or third-level educat* or universit*).ab. or (college* or graduate educat* or higher educat* or post-secondary educat* or tertiary educat* or third-level educat* or universit*).ti.	106105
2	exp universities/	4247
3	exp higher education/	1235
4	(agenda 2030 or SDG or smart growth or sustainability educat* or sustainable develop* or sustainble develop*).ab. or (agenda 2030 or SDG or smart growth or sustainability educat* or sustainable develop* or sustainble develop*).ti.	6030
5	((UN or united nation*) adj3 development goal*).ab. or ((UN or united nation*) adj3 development goal*).ti.	555
6	1 or 2 or 3	106595
7	4 or 5	6222
8	6 and 7	203
**Search strategy for ERIC database**		
Interface: ProQuest Date of Search: 6^th^ Oct 2021 Number of hits: 1883		
#1	MAINSUBJECT.EXACT.EXPLODE(‘Sustainable Development’)	4,345
#2	TI,AB(‘agenda 2030’ OR ‘SDG’ OR ‘smart growth’ OR ‘sustainability educat*’ OR ‘sustainable develop*’ OR ‘sustainble develop*’)	2,954
#3	TI,AB((‘UN’ OR ‘united nation*’) NEAR/3 ‘development goal*’)	140
#4	MAINSUBJECT.EXACT.EXPLODE(‘Higher Education’)	310,656
#5	MAINSUBJECT.EXACT.EXPLODE(‘Universities’)	34,030
#6	TI,AB(college* or ‘graduate educat*’ or ‘higher educat*’ or ‘post-secondary educat*’ or ‘tertiary educat*’ or ‘third-level educat*’ or universit*)	394,673
#7	#1 or #2 or #3	5110
#8	#4 or #5 or #6	529,036
#9	#7 AND #8	1883

## References

[1] United Nations. Department of Economic and Social Affairs. Sustainable development. THE 17 GOALS | sustainable development [Internet]; 2015 [cited 2022 Apr 19]. Available from: https://sdgs.un.org/goals

[2] Takian A, Akbari-Sari A. Sustainable health development becoming agenda for public health academia. Iran J Public Health. 2016;45:1502–14. 28028502 PMC5182259

[3] Mawonde A, Togo M. Implementation of SDGs at the University of South Africa. Int J Sustain High Educ. 2019;20:932–950.

[4] Global indicator framework for the sustainable development goals and targets of the 2030 Agenda for sustainable development. Work Stat Comm Pertain to 2030 Agenda Sustain Dev [Internet]. 2020;1–21. Available from: https://unstats.un.org/sdgs/indicators/GlobalIndicatorFrameworkafter2019refinementEng.pdf%0A

[5] Sustainable Development Solutions Network, Autralia/Pacific. Getting started with the SDGs in Universities. A guide for Universities, higher education institutions, and the academic sector. Australia, New Zealand and Pacific Edition. [Internet]; 2017. Available from: http://ap-unsdsn.org/wp-content/uploads/2017/08/University-SDG-Guide_web.pdf

[6] Maeshiro R, Johnson I, Koo D, Parboosingh J, Carney JK, Gesundheit N, et al. Medical education for a healthier population: reflections on the Flexner report from a public health perspective. Acad Med. 2010;85:211–219. 20107345 10.1097/ACM.0b013e3181c885d8

[7] Wersun A, Azmat F, Suri H, Hauser C. Blueprint for SDG integration into curriculum, research and partnerships. [Internet]; 2020. Available from: https://d30mzt1bxg5llt.cloudfront.net/public/uploads/PDFs/BlueprintForSDGIntegration.pdf

[8] Sustainable Development Solutions Network. Accelerating Education for the SDGs in Universities: a guide for universities, colleges, and tertiary and higher education institutions [Internet]; 2020 [cited 2022 Apr 19]. Available from: https://resources.unsdsn.org/accelerating-education-for-the-sdgs-in-universities-a-guide-for-universities-colleges-and-tertiary-and-higher-education-institutions

[9] Cambridge English Dictionary: Meanings & Definitions [Internet]; 2022 [cited 2022 Apr 21]. Available from: https://dictionary.cambridge.org/dictionary/english/

[10] Clifford KL, Zaman MH. Engineering, global health, and inclusive innovation: focus on partnership, system strengthening, and local impact for SDGs. Glob Health Action. 2016;9:30175. 26790462 10.3402/gha.v9.30175 PMC4720687

[11] Craveiro I, Carvalho AA, Ferrinho P. “Get us partnerships!” - A qualitative study of Angolan and Mozambican health academics’ experiences with North/South partnerships. Global Health. 2020;16:33. 32295611 10.1186/s12992-020-00562-7 PMC7161017

[12] Arksey H, O’malley L. Scoping studies: towards a methodological framework. Int J Soc Res Methodol Theory Pract. 2005;8:19–32.

[13] Tricco AC, Lillie E, Zarin W, O’brien KK, Colquhoun H, Levac D, et al. PRISMA extension for scoping reviews (PRISMA-Scr): checklist and explanation. Ann Intern Med. 2018;169:467–473. 30178033 10.7326/M18-0850

[14] Peters MDJ, Marnie C, Tricco AC, Pollock D, Munn Z, Alexander L, et al. Updated methodological guidance for the conduct of scoping reviews. JBI Evid Synth. 2020;18:2119–2126. 33038124 10.11124/JBIES-20-00167

[15] Page MJ, McKenzie JE, Bossuyt PM, Boutron I, Hoffmann TC, Mulrow CD, et al. The PRISMA 2020 statement: an updated guideline for reporting systematic reviews. BMJ. 2021;372. DOI:10.1136/bmj.n71. PMC8005924 33782057

[16] United Nations. United Nations iLibrary [Internet]; 2022 [cited 2022 Jun 22]. Available from: https://www.un-ilibrary.org/

[17] UNESCO. [Internet]; 2022 [cited 2022 Jun 22]. Available from: https://www.unesco.org/en/brief

[18] IAU - International Association of Universities. [Internet]; 2022 [cited 2022 Jun 22]. Available from: https://www.iau-aiu.net/

[19] ShanghaiRanking. [Internet]; 2021 [cited 2022 Jun 22]. Available from: https://www.shanghairanking.com/

[20] QS World University Rankings. [Internet]; 2021 [cited 2022 Jun 22]. Available from: https://www.topuniversities.com/

[21] Times Higher Education [Internet]; 2022 [cited 2022 Jun 22]. Available from: https://www.timeshighereducation.com/world-university-rankings

[22] Rayyan – intelligent systematic review [Internet]. [cited 2022 Apr 19]. Available from: https://www.rayyan.ai/

[23] The World Bank. World Bank Country and Lending Groups [Internet]; 2021 [cited 2022 Jun 20]. Available from: https://datahelpdesk.worldbank.org/knowledgebase/articles/906519-world-bank-country-and-lending-groups

[24] Blasco N, Brusca I, Labrador M. Drivers for universities’ contribution to the sustainable development goals: an analysis of Spanish Public universities. Sustainability. 2021;13:1–19.

[25] Purcell WM, Henriksen H, Spengler JD. Universities as the engine of transformational sustainability toward delivering the sustainable development goals: “living labs” for sustainability. Int J Sustain High Educ. 2019;20:1343–1357.

[26] Useh U. Sustainable development goals as a framework for postgraduate future research following COVID-19 pandemic: a new norm for developing countries. High Educ Futur. 2021;8:123–132.

[27] Ezquerra I, Gómez A, Mataix C, Soberón M, Moreno J, Sánchez T. A dialogical approach to readiness for change towards sustainability in higher education institutions: the case of the sdgs seminars at the universidad politécnica de Madrid. Sustainability. 2021;13:9168.

[28] Da Wan C, Abdullah D. Internationalisation of Malaysian higher education: policies, practices and the SDGs. Int J Comp Educ Dev. 2021;23:212–226.

[29] Togo M, Gandidzanwa CP. The role of Education 5.0 in accelerating the implementation of SDGs and challenges encountered at the University of Zimbabwe. Int J Sustain High Educ. 2021;22:1520–1535.

[30] Hansen B, Stiling P, Uy WF. Innovations and challenges in SDG integration and reporting in higher education: a case study from the University of South Florida. Int J Sustain High Educ. 2021;22:1002–1021.

[31] Petillion RJ, Freeman TK, McNeill WS. United Nations sustainable development goals as a thematic framework for an introductory chemistry curriculum. J Chem Educ. 2019;96:2845–2851.

[32] Albareda S, Vidal S, Fernández M. Implementing the sustainable development goals at university level. Int J Sustain High Educ. 2018;19:473–497.

[33] Aleixo AM, Azeiteiro UM, Leal S. Are the sustainable development goals being implemented in the Portuguese higher education formative offer? Int J Sustain High Educ. 2020;21:336–352.

[34] Anasi SN, Ukangwa CC, Fagbe A. University libraries-bridging digital gaps and accelerating the achievement of sustainable development goals through information and communication technologies. World J Sci Technol Sustain Dev. 2018;15:13–25.

[35] Chaleta E, Saraiva M, Leal F, Fialho I, Borralho A. Higher education and sustainable development goals (Sdg)—potential contribution of the undergraduate courses of the school of social sciences of the university of évora. Sustainability. 2021;13:1–10.

[36] Chang YC, Lien HL. Mapping course sustainability by embedding the SDGs inventory into the university curriculum: a case study from National University of Kaohsiung in Taiwan. Sustainability. 2020;12:4274.

[37] Cottafava D, Cavaglia G, Corazza L, Cavaglià G, Corazza L. Education of sustainable development goals through students’ active engagement a transformative learning experience. Sustain Account Manag Policy J. 2019;10:521–544.

[38] Exposito LMC, Sanchez JG. Implementation of SDGs in university teaching: a course for professional development of teachers in education for sustainability for a transformative action. Sustainability. 2020;12:8267.

[39] Leal W, Shiel C, Paco A, Mifsud M, Avila LV, Brandli LL, et al. Sustainable development goals and sustainability teaching at universities: falling behind or getting ahead of the pack? J Clean Prod. 2019;232:285–294.

[40] Lovren VO, Maruna M, Stanarevic S, Orlovic Lovren V, Maruna M, Stanarevic S. Reflections on the learning objectives for sustainable development in the higher education curricula – three cases from the University of Belgrade. Int J Sustain High Educ. 2020;21:315–335.

[41] Ramirez RA, Morales R, Melchor EM, Iqbal HMN, Parrao L, Vargas A, et al. Incorporating the sustainable development goals in engineering education. IJIDEM. 2020;14:739–745.

[42] Gómez ME, Gimenez E, Andrés I, Pellicer E, Gomez ME. Boosting the sustainable development goals in a civil engineering bachelor degree program. Int J Sustain High Educ. 2021;22:125–145.

[43] UNESCO. SDG 4 data digest 2021 national SDG 4 benchmarks: fulfulling our neglected commitment. [Internet]. 2021. Available from: https://unesdoc.unesco.org/in/documentViewer.xhtml?v=2.1.196&id=p:usmarcdef_0000380387&file=/in/rest/annotationSVC/DownloadWatermarkedAttachment/attach_import_b6603b17-86ad-4c5c-821d-de2a61cc637f%3F_%3D380387eng.pdf&locale=es&multi=true&ark=/ark:/48223/p

[44] UNESCO. Education 2030: Incheon declaration and framework for action for the implementation of sustainable development goal 4: ensure inclusive and equitable quality education and promote lifelong learning opportunities for all [Internet]; 2016. Available from: https://unesdoc.unesco.org/ark:/48223/pf0000245656

[45] International Association of Universities: Higher Education for Sustainable Development. Amrita University. Amrita Center for Sustainable Future [Internet]; 2018 [cited 2022 Jun 22]. Available from: http://www.iau-hesd.net/actions/4131/amrita-center-sustainable-future

[46] International Association of Universities: Higher Education for Sustainable Development. Beirut Arab University. SDG actions and social impact [Internet]; 2021 [cited 2022 Jun 22]. Available from: http://www.iau-hesd.net/actions/5060/beirut-arab-university-sdg-actions-and-social-impact

[47] International Association of Universities: Higher Education for Sustainable Development. University of Management and Technology, Lahore. BS Energy Engineering [Internet]; 2018 [cited 2022 Jul 18]. Available from: http://www.iau-hesd.net/actions/4315/bs-energy-engineering

[48] International Association of Universities: Higher Education for Sustainable Development. Toyo University. Center for Sustainable Development Studies [Internet]; 2018 [cited 2022 Jun 22]. Available from: http://www.iau-hesd.net/actions/4255/center-sustainable-development-studies

[49] University of Cape Town.UCT and the UN Sustainable Development Goals [Internet]; 2022 [cited 2022 Jul 18]. Available from: https://www.uct.ac.za/main/research/sdgs

[50] University of Johannesburg. Sustainable Development Goals Archive [Internet]; 2021. [cited 2022 Jul 18]. Available from: https://www.uj.ac.za/sdg/

[51] Cairo University. [Internet]; 2017 [cited 2022 Jul 18]. Available from: https://cu.edu.eg/Home

[52] Addis Ababa University. [Internet]; 2022 [cited 2022 Jul 18]. Available from: http://graduation.aau.edu.et/

[53] National University of Singapore. [Internet]; 2022 [cited 2022 Jul 18]. Available from: https://www.nus.edu.sg/

[54] The University of Tokyo. [Internet]; 2022 [cited 2022 Jul 18]. Available from: https://www.u-tokyo.ac.jp/en/

[55] International Association of Universities: Higher Education for Sustainable Development. College of Environmental Engineering and Architecture [Internet]; 2021 [cited 2022 Jul 18]. Available from: https://www.iau-hesd.net/actions/4218/college-environmental-engineering-and-architecture

[56] Seoul National University. [Internet]; 2022 [cited 2022 Jun 22]. Available from: https://en.snu.ac.kr/

[57] University of Science and Technology of China. [Internet]; 2022 [cited 2022 Jul 18]. Available from: https://en.ustc.edu.cn/

[58] University of Oxford. [Internet]; 2022 [cited 2022 Jul 18]. Available from: https://www.ox.ac.uk/

[59] ETH Zurich. [Internet]; 2022 [cited 2022 Jun 22]. Available from: https://ethz.ch/en.html

[60] Université PSL. [Internet]; 2022 [cited 2022 Jul 18]. Available from: https://psl.eu/en

[61] University of Copenhagen. [Internet]; 2022 [cited 2022 Jul 18]. Available from: https://www.ku.dk/english/

[62] LMU Munich. [Internet]; 2022 [cited 2022 Jul 18]. Available from: https://www.lmu.de/en/

[63] Universidad de Chile. [Internet]; 2022 [cited 2022 Jul 18]. Available from: https://www.uchile.cl/

[64] Tecnológico de Monterrey. [Internet]; 2022 [cited 2022 Jul 18]. Available from: https://tec.mx/es

[65] The University of Chicago. [Internet]; 2022 [cited 2022 Jul 18]. Available from: https://www.uchicago.edu/

[66] International Association of Universities: Higher Education for Sustainable Development. University of Bergen. The University of Bergen launches of a new series of SDG policy briefs. [Internet]; 2021 [cited 2022 Jun 22]. Available from: http://www.iau-hesd.net/actions/5223/university-bergen-launches-new-series-sdg-policy-briefs

[67] Columbia University in the City of New York. [Internet]; 2022 [cited 2022 Jul 18]. Available from: https://www.columbia.edu/

[68] Brown University. [Internet]; 2022 [cited 2022 Jul 18]. Available from: https://www.brown.edu/

[69] The University of Melbourne. [Internet]; 2022 [cited 2022 Jul 18]. Available from: https://www.unimelb.edu.au/

[70] The University of Queensland. [Internet]; 2022 [cited 2022 Jul 18]. Available from: https://www.uq.edu.au/

[71] University of Technology Sydney. [Internet]; 2022 [cited 2022 Jul 18]. Available from: https://www.uts.edu.au/

[72] Curtin University [Internet]; 2022 [cited 2022 Jul 18]. Available from: https://www.curtin.edu.au/

[73] QUT University. [Internet]; 2022 [cited 2022 Jul 18]. Available from: https://www.qut.edu.au/

[74] Harvard University. [Internet]; 2022 [cited 2022 Jul 18]. Available from: https://www.harvard.edu/

[75] International Association of Universities: Higher Education for Sustainable Development. Fish in Sustainable Food Systems program at the University of Wollongong [Internet]; 2021 [cited 2022 Jul 18]. Available from: http://www.iau-hesd.net/actions/5166/fish-sustainable-food-systems-program-university-wollongong

[76] International Association of Universities: Higher Education for Sustainable Development. University of Oslo. Achieving the SDGs: Global goals and national interests [Internet]; 2020 [cited 2022 Jul 18]. Available from: http://www.iau-hesd.net/actions/4917/achieving-sdgs-global-goals-and-national-interests

[77] International Association of Universities. The University of the West Indies. A community-centred approach to education for sustainable development [Internet]; 2017 [cited 2022 Jun 22]. Available from: http://www.iau-hesd.net/actions/1177/community-centred-approach-education-sustainable-development

[78] International Association of Universities. Higher education for sustainable development. The association of common wealth universities. ACU summer school 2022. [Internet]; 2022 [cited 2022 Jul 18]. Available from: http://www.iau-hesd.net/actions/5515/acu-summer-school-2022-sport-and-regeneration-driving-sustainability-community-and

[79] International Association of Universities: Higher Education for Sustainable Development. Fernando Pessoa University. Architecture and Urbanism (integrated master’s degree) [Internet]; 2018 [cited 2022 Jul 18]. Available from: http://www.iau-hesd.net/actions/4335/architecture-and-urbanism-integrated-masters-degree

[80] Wan CD, Abdullah D. Internationalisation of Malaysian higher education: policies, practices and the SDGs. Int J Comp Educ Dev [InternetAvailable from]. 2021;23:212–226.

[81] Sustainable Development Solutions Network. Accelerating education for the SDGs in Universities: save the planet through communication [Internet]; 2021 [cited 2022 May 4]. Available from: https://blogs.upm.es/education4sdg/2021/08/11/save-the-planet-through-communication/

[82] Wals AEJ. Sustainability-oriented ecologies of learning: a response to systemic global dysfunction. Ecol Learn Pract. 2019;5:61–78.

[83] Lozano R. Are companies planning their organisational changes for corporate sustainability? An analysis of three case studies on resistance to change and their strategies to overcome it. Corp Soc Responsib Environ Manag. 2013;20:275–295.

[84] Global University Network for Innovation. 2nd GUNi International Conference on SDGs: Higher Education & Science Take Action. Barcelona; 2020.

[85] Al-Mandhari A, El-Adawy M, Khan W, Ghaffar A. Health for all by all-pursuing multi-sectoral action on health for SDGs in the WHO Eastern Mediterranean region. Global Health. 2019;15:1–4. 31847852 10.1186/s12992-019-0504-8 PMC6918621

[86] Chotchoungchatchai S, Marshall AI, Witthayapipopsakul W, Panichkriangkrai W, Patcharanarumol W, Tangcharoensathien V. Primary health care and sustainable development goals. Bull World Health Organ. 2020;98:792. 33177776 10.2471/BLT.19.245613 PMC7607463

[87] Safari A, Hosseini Z. The effect of workshop training method and electronic teaching method on mathematics learning. Eur J Multidiscip Stud. 2016;3:165.

[88] Grindsted TS, Theis Nielsen T. Spaces of learning-practising the SDGs through geographical fieldwork methods in a nature park. Int J Sustain High Educ. 2022;23:105–119.

[89] Garbati J, Samuels B. Publishing in educational research journals: are graduate students participating? J Sch Publ [Internet]; 2013 Jul 1 [cited 2023 Feb 20];44:355–372. Available from: https://www.researchgate.net/publication/265893039_Publishing_in_Educational_Research_Journals_Are_Graduate_Students_Participating

[90] Binagwaho A, Allotey P, Sangano E, Ekström AM, Martin K. A call to action to reform academic global health partnerships. BMJ. 2021;375:2658. 10.1136/bmj.n2658 34725093

[91] Sewankambo N, Tumwine JK, Tomson G, Obua C, Bwanga F, Waiswa P, et al. Enabling dynamic partnerships through joint degrees between low- and high-income countries for capacity development in global health research: experience from the Karolinska Institutet/Makerere university partnership. PLOS Med. 2015;12:e1001784. 25646629 10.1371/journal.pmed.1001784 PMC4315568

[92] Karolinska Institutet. Centre of Excellence for Sustainable Health. [Internet]; 2022 [cited 2022 Sep 14]. Available from: https://ki.se/en/collaboration/centre-of-excellence-for-sustainable-health

[93] Vergnes JN, Marchal-Sixou C, Nabet C, Maret D, Hamel O. Ethics in systematic reviews. J Med Ethics. 2010;36:771–774. 20952493 10.1136/jme.2010.039941

[94] Suri H. Ethical considerations of conducting systematic reviews in educational research. Syst Rev Educ Res. 2020;41–54.

[95] Tricco AC, Lillie E, Zarin W, O’brien KK, Colquhoun H, Levac D, et al. Preferred reporting items for systematic reviews and meta-analyses extension for scoping reviews (PRISMA-Scr). Ann Intern Med. 2018;169:11–12. 10.7326/M18-0850 30178033

